# Inflammatory Bowel Disease and Cardiovascular Disease: An Integrative Review With a Focus on the Gut Microbiome

**DOI:** 10.7759/cureus.65136

**Published:** 2024-07-22

**Authors:** Camila Sanchez Cruz, Anahi Rojas Huerta, Jesus Lima Barrientos, Cristina Rodriguez, Aarfa Devani, Vanessa Boosahda, Naga S Rasagna Mareddy, Gabriela Briceno Silva, Jose C Del Castillo Miranda, Kevin A Reyes Gochi, Mario D Reyes Gochi, Samantha Alvarez, Patricia E Ghattas Hasbun

**Affiliations:** 1 General Practice, Universidad Nacional Autonoma de Mexico, Mexico City, MEX; 2 General Practice, Benemérita Universidad Autónoma de Puebla, Puebla, MEX; 3 Internal Medicine, RWJBarnabas Health Community Medical Center, Toms River, USA; 4 General Practice, Malla Reddy Institute of Medical Sciences, Hyderabad, IND; 5 General Practice, Xavier University School of Medicine, Oranjestad, ABW; 6 Radiology, University of Alabama at Birmingham, Birmingham, USA; 7 Obstetrics and Gynaecology, Universidad de Oriente, Barcelona, VEN; 8 General Practice, Universidad Peruana Cayetano Heredia, San Martín de Porres, PER; 9 School of Medicine, Universidad Nacional Autonoma de Mexico, Mexico City, MEX; 10 Surgery, Universidad Nacional Autonoma de Mexico, Mexico City, MEX; 11 General Practice, St. George’s University, West Indies, GRD; 12 General Practice, Universidad Católica de Honduras, Tegucigalpa, HND

**Keywords:** dysbiosis and endothelial dysfunction, chronic systemic inflammation, gut microbiome, cardiovascular disease (cvd), inflammatory bowel disease (ibd)

## Abstract

Inflammatory bowel disease (IBD), which includes Crohn's disease and ulcerative colitis, is a chronic inflammatory condition of the gastrointestinal tract. Recent research indicates a significant link between IBD and cardiovascular disease (CVD), the leading cause of global morbidity and mortality. This review examines the association between IBD and CVD, emphasizing the role of the gut microbiome in this relationship. IBD patients have a higher risk of cardiovascular events, such as coronary artery disease, heart failure, and cerebrovascular incidents, primarily due to chronic systemic inflammation, genetic factors, and gut microbiota imbalance (dysbiosis). Dysbiosis in IBD increases intestinal permeability, allowing bacterial products to enter the bloodstream, which promotes inflammation and endothelial dysfunction, contributing to CVD.

Understanding the gut microbiome's role in IBD and CVD suggests new therapeutic interventions. Modulating the microbiome through diet, probiotics, and fecal microbiota transplantation (FMT) are promising research avenues. These interventions aim to restore a healthy gut microbiota balance, potentially reducing inflammation and improving cardiovascular outcomes. Additionally, the review emphasizes the importance of regular cardiovascular risk assessments and personalized preventive measures in managing IBD patients. Such measures include routine monitoring of cardiovascular health, tailored lifestyle modifications, and early intervention strategies to mitigate cardiovascular risk. By integrating current knowledge, this review aims to improve understanding and management of the interconnected pathophysiology of IBD and CVD. This approach will ultimately enhance patient outcomes and provide a foundation for future research and clinical practice guidelines in this area.

## Introduction and background

Inflammatory bowel disease (IBD) is a digestive system disorder of multifactorial etiology characterized by chronic inflammation in the gastrointestinal tract. It is divided into ulcerative colitis (UC) and Crohn's disease (CD), which, despite their similarity, have differences in their symptomatology. Epidemiologically, both spectrums of the disease can occur in both adults and children, and geographic location may affect the prevalence and incidence of the disease. The importance of its detection and management lies in the risk of complications, extraintestinal manifestations, and deterioration in the quality of life they cause in patients [1]. Currently, cardiovascular diseases (CVD) continue to be the leading cause of mortality and morbidity worldwide, the most common of which are coronary heart disease and cerebrovascular disease. In recent years, it has been discovered that the pathophysiology of cardiovascular diseases goes beyond a process of local endothelial damage, where diverse mechanisms of inflammation intervene and generate links with chronic inflammatory diseases [2]. Because of this, several studies have already shown that there is an association between IBD and a higher incidence of cardiovascular events, apparently due to the increased cardiovascular risk that these patients present [3]. The relationship between these entities is of utmost importance as it represents an increased risk of flares, persistence of disease activity, and death from cardiovascular disease [4]. 

In recent years, the microbiome has been extensively studied to such an extent that its description has changed. However, the original description as a characteristic microbial community in a reasonably well-defined habitat with distinct physicochemical properties as its theater of activity is still the most consensually adequate [5]. The relationship between IBD and the microbiome has been proven in the literature, with dysbiosis being a fundamental basis for pathophysiological development by being an inducer of inflammation and metabolic alterations [6]. Due to the above, the intestinal microbiome and its role in these inflammatory diseases must be given due importance. It has possible diagnostic and therapeutic potential that could lead to better understanding and treating the cardiovascular diseases associated with BD. This article aims to provide an updated and reliable review that will allow the medical community to understand the relationship between IBD and CVD, taking into account the implications of the gut microbiome.

## Review

Epidemiology of IBD and CVD

It has been calculated that approximately six million patients have IBD worldwide, with approximately half of those cases in the US alone. Patients diagnosed with IBD have an increased risk of several cardiovascular diseases, including but not limited to illnesses such as coronary heart disease and stroke. For example, one study showed that after adjusting for traditional CV risk factors, patients with IBD have two times an increase in the risk of heart failure compared to patients who do not have IBD [7]. When looking at the diseases that make up IBD separately, Crohn's disease and ulcerative colitis, we see even more distinct pathways to heart disease from the mechanisms of each disease. Crohn's disease is itself associated with an increased risk of atherosclerosis, cerebrovascular accidents, premature coronary artery disease, and atrial fibrillation. Additionally, ulcerative colitis has been associated with an increased risk of heart failure, specifically during an IBD flare-up, but not during periods of remission. This provides evidence that there is a direct link between active inflammatory disease and the sequelae of cardiovascular damage.

Patients with IBD tend to see CVD in earlier decades of life than the rest of the population. It has been described that CVD risk is higher in young adults than older adults in patients with IBD. There is also evidence that shows that patients with IBD have a 19% increase in their risk of developing heart failure up to 20 years after diagnosis, regardless of what type of IBD the patient had [8]. In patient populations where CVD is already higher, such as in pregnant patients, those who were pregnant and had IBD showed evidence of a two to three times increased risk of VTE throughout the pregnancy and even spanning into the postpartum period [9]. 

Genetic risk factors implicated in CVD development in patients with inflammatory bowel disease include NOD2 mutation, ATG16L1 deficiency, IL-10 mutation, RAG2 deficiency, and NEMO deletion. These mutations, which cause IBD and gut dysbiosis, have been shown to contribute to a proinflammatory state in the IBD patient's vessels, leading to a variety of atherosclerotic pathologies. There is also evidence that alterations in the gut microbiome composition can affect a patient's CVD risk. This occurs because as people age and the new industrialized society becomes obese, sedentary, or changes their dietary patterns, intestinal inflammation occurs. These factors are compounded in a patient who has IBD. As the inflammation seen in a patient with IBD during an acute flair of their disease proliferates, it compromises the gut barrier. As the barrier is compromised, the intestinal barrier is altered in function and permeability, which results in the translocation of bacteria and their bacterial products into the systemic circulation. Herein lies the connection between the increase in proinflammatory diseases and CVD [10]. There is evidence that certain drugs, particularly biological TNF-alpha inhibitors, which are commonly used in patients with IBD, have been associated with an increase in the risk of venous thromboembolism (VTE) through the promotion of endothelial dysfunction and thrombus formation [11].

Pathophysiology of IBD and its link to CVD 

Pathophysiologically, IBD is complex as it involves the interaction between four components: an aberrant immune system, genetic factors, environmental factors, and the intestinal microbiota that result in chronic inflammation. The inflammatory response is mainly mediated by T-helper 1 and T-helper 17 cells in the case of Chron's disease and T-helper 2 in the case of ulcerative colitis, in addition to cytokines such as tumor necrosis factor-α (TNF-α), transforming growth factor-β, interleukines, reactive oxygen species (ROS), neuropeptides and non-immune cells [12]. Derived from the above, when there is a primary immune reaction to one or more stimuli, tissue destruction and proliferation of endothelial and mesenchymal cells are induced, leading to a secondary immune response amplifying the inflammation already present and stimulating fibrosis, tissue remodeling, angiogenesis, and lymphangiogenesis. If this inflammation is not solved, it determines the installation of a vicious circle of chronic self-sustained inflammation and maintenance of angiogenesis, fibrosis, and tissue destruction processes [13].

Upon the establishment of a local and systemic inflammatory state in IBD, several inflammatory biomarkers, including C-reactive protein (CRP), serum amyloid A (SAA), TNF-α, interleukin IL-1β, IL-6, IL-8, IL-12, and calprotectin, increase significantly. In fact, these markers are associated with CVD, of which CRP and SAA are related to increased cardiovascular risk and mortality. The previously mentioned interleukins are associated with endothelial dysfunction and coronary artery disease, while calprotectin is associated with atherosclerosis [14]. The evident relationship in the mechanism of inflammation suggests that there are shared pathways between both pathologies, involving several key factors for the pathogenesis of these diseases [15]. 

One factor strongly implicated with CVD is dysfunction of the endothelium, a single layer of epithelium that represents the inner surface of the cardiovascular system and whose main function is to maintain vascular homeostasis by producing a series of mediators such as nitric oxide (NO), prostacyclin, endothelin, von Willebrand factor, and cell adhesion molecules. Their dysfunction, induced by a chronic inflammatory state such as that occurring in IBD, is a state in which the balance between vasodilator and vasoconstrictor factors is altered, leading to increased expression of cell adhesion molecules, barrier malfunction with increased leukocyte diapedesis, elevated smooth muscle tone due to decreased production of vasodilator substances and increased production of vasoconstrictor substances [16]. In fact, the most potent vasodilator molecule, NO, is found to be decreased in patients with IBD since they have an increase in TNF-alpha and a higher expression of the enzyme arginase, which competes for L-arginine that is catalyzed by endothelial NO synthase [17]. Furthermore, it is important to highlight that endothelial damage leads to the development of angiogenesis, which has been shown to play a role in the maintenance of intestinal inflammation mainly through the CD40-CD40 ligand pathway, leading to the secretion of proangiogenic cytokines [17-19]. 

Pathological vascular reactivity derived from a state of chronic inflammation contributes to the formation of pathogenic events within the vessel wall, such as atherosclerosis. Even during atherosclerotic plaque formation, the TH 1 response and INF-γ production lead to an increase in TNF-α, Il-1, Il-6, and CRP that, as a consequence, produce additional inflammatory and cytotoxic molecules [20]. However, multiple processes have been implicated in the pathogenesis of atherosclerotic cardiovascular disease (ACVD), in addition to inflammation and endothelial dysfunction, such as thrombosis, lipid dysfunction, harmful effects of some IBD therapies and, more importantly in recent years, the imbalance of the intestinal microbiome [21]. 

**Figure 1:**
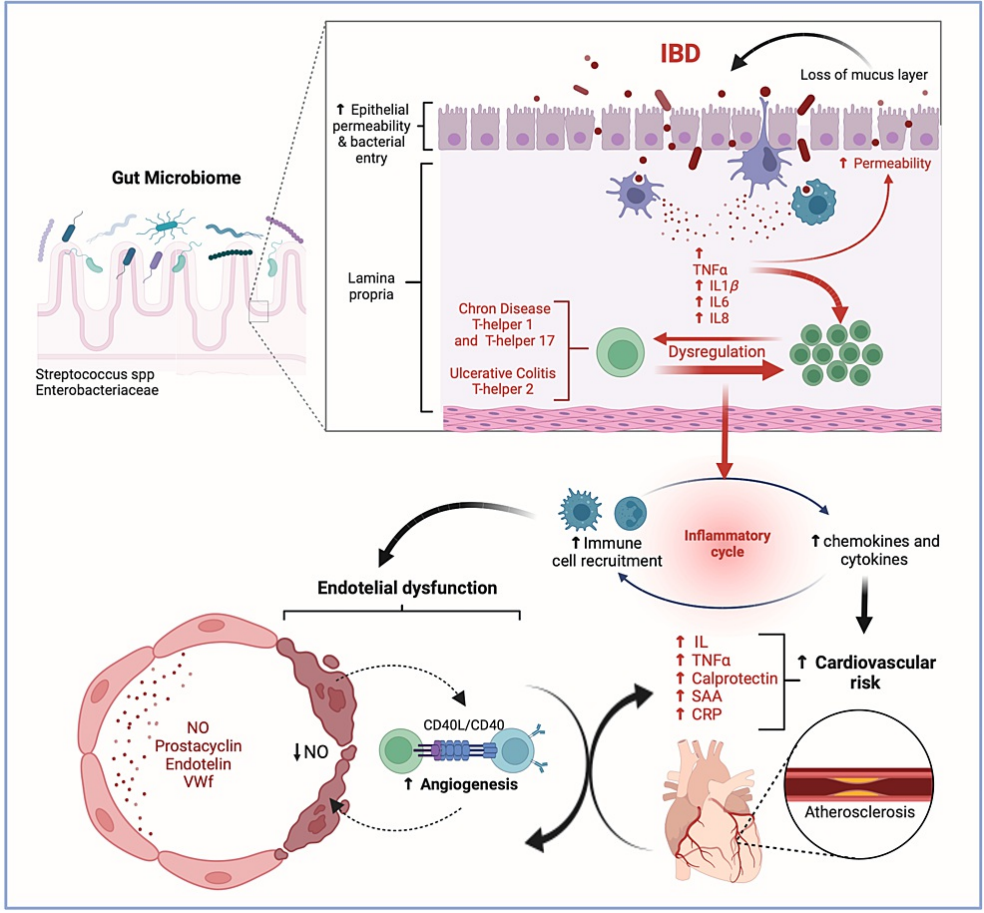
Pathophysiological links between inflammatory bowel disease and cardiovascular disease. Interaction between gut microbiota, immune dysregulation in IBD, and its effects on cardiovascular disease. Increased intestinal permeability and bacterial entry in IBD lead to chronic inflammation mediated by T-helper cells and cytokines. This chronic inflammation and endothelial dysfunction contribute to cardiovascular risks, including atherosclerosis, driven by factors like TNF-α, IL-6, and CRP. Figure created with BioRender. All credits to Anahi Rojas Huerta. IBD: inflammatory bowel disease, TNF-α: tumor necrosis factor-alpha, IL1β: interleukin 1 beta, IL6: interleukin 6, and IL8: interleukin 8, NO: nitric oxide, VWf: von Willebrand factor, CD40L: CD40 ligand, spp: Streptococcus species, CRP: C-reactive protein, SAA: serum amyloid A.

Intestinal dysbiosis, which is defined as an imbalance in the microbiome, has recently been defined as an important risk factor for the development of IBD and CVD [22]. Examples of this are the alterations in the Firmicutes/Bacteroidetes ratio associated with hypertension and the enrichment in Streptococcus spp.-Enterobacteriaceae, including *Escherichia coli*, which is observed in patients with IBD and CVD [23-25]. Also, this imbalance may increase intestinal permeability, leading to elevated absorption of lipopolysaccharide (LPS) in the intestines, which in turn increases the secretion of proinflammatory cytokines, exacerbating atherosclerosis, inducing macrophage activation, vascular endothelialitis and increasing CRP [25]. Intestinal bacterial metabolites, such as indole and phenyl derivatives, also exacerbate atherosclerosis and cause hypertension. In addition, dysbiosis-induced metabolic alterations, in particular increased production of trimethylamine N-oxide (TMAO), have been implicated in elevating cardiovascular risk through proinflammatory cytokine expression, low-density lipoprotein (LDL) deposition, and cardiac mitochondrial dysfunction. TMAO promotes foam cell formation, endothelial dysfunction, and platelet activation, thus contributing to plaque formation and thrombosis [26]. In contrast, normal intestinal flora produces beneficial short-chain fatty acids (SCFA), such as butyrate and propionate, which exhibit anti-inflammatory properties and may confer protection against cardiovascular disease [27] (Figure 1).

The study of the interconnections in the pathophysiology of IBD and CVD remains a challenge, and further research is needed to determine reliable risk markers and discover pathways that increase the likelihood of comprehensive management in these patients.

Gut microbiome and its role in IBD and CVD 

A growing body of evidence indicates that gut microbiota composition significantly influences cardiovascular health conditions, including hypertension, atrial fibrillation, and heart failure. The gut microbiota plays a crucial role in cardiovascular health. Dietary interventions targeting the gut microbiome have shown promise in enhancing cardiovascular outcomes. For example, high-quality carbohydrates can positively influence fecal microbiota, suggesting that specific dietary changes can improve cardiometabolic health. This opens new possibilities for dietary interventions to modulate the gut microbiota to boost cardiovascular health [28].

Changes in the gut microbiome are also linked to blood pressure regulation. Hypertension has been associated with significant shifts in gut microbial communities [29]. This suggests that targeting the gut microbiota might offer a novel approach to managing hypertension, complementing traditional treatments. The gut microbiome also affects the risk of atrial fibrillation (AF). Research using extensive genetic datasets has shown that certain gut microbes can influence the risk of developing AF. This highlights potential therapeutic interventions that could target these specific gut microbes to manage or prevent AF, including probiotics, prebiotics, dietary modifications, fecal microbiota transplantation (FMT), pharmacological agents, genetic approaches, and personalized medicine [30].

**Figure 2:**
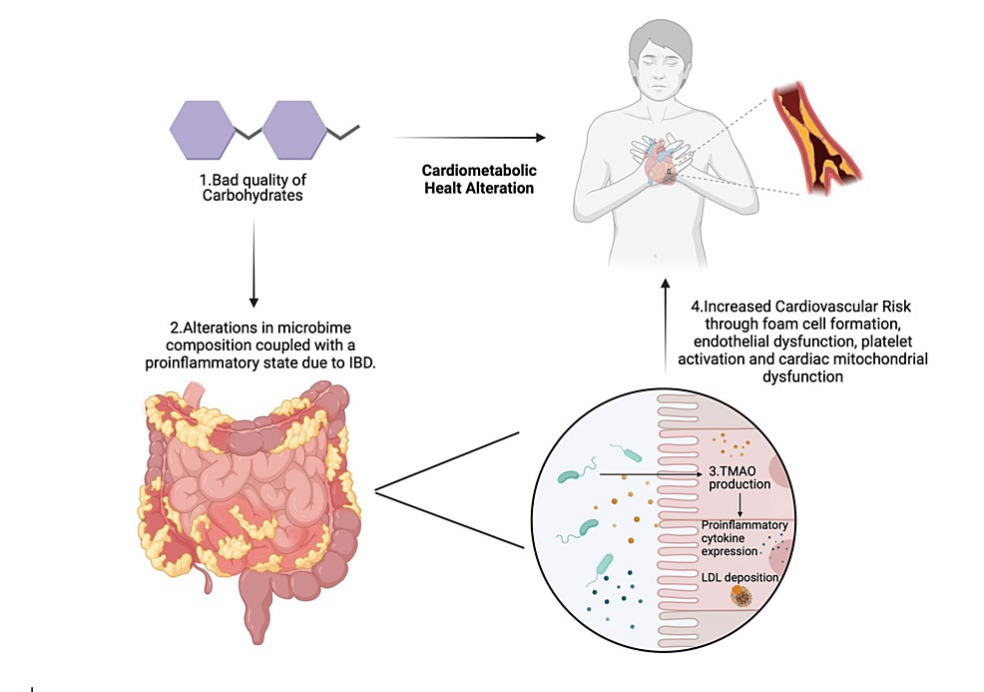
Role of diet and the microbiome in IBD and cardiometabolic disorders. This diagram illustrates the relationship between gut microbiome alterations and cardiovascular health. Poor-quality carbohydrates lead to changes in gut microbiota composition and a proinflammatory state in IBD. This triggers the production of TMAO, a metabolite linked to increased cardiovascular risk. Elevated TMAO levels contribute to foam cell formation, endothelial dysfunction, platelet activation, and cardiac mitochondrial dysfunction, thereby increasing the risk of cardiovascular diseases. Figure created with BioRender. All credits to Jesus Lima Barrientos. LDL: low-density lipoprotein, IBD: inflammatory bowel disease, TMAO: trimethylamine N-oxide.

TMAO, a key metabolite produced by gut bacteria, has been widely studied for its role in cardiovascular health. Elevated TMAO levels are linked to a higher risk of CVD, including heart attacks and strokes [31]. This connection highlights the importance of managing TMAO levels through dietary and microbial interventions. Reducing the intake of TMAO precursors found in certain foods or using probiotics and prebiotics to influence gut bacteria that produce TMAO can be effective strategies to lower cardiovascular risk. The link between gut health and cardiovascular conditions, such as ischemic stroke and heart failure, further emphasizes the vital role of the gut microbiome. Poor gut health can significantly increase stroke risk, underscoring the need to maintain a healthy gut microbiome to prevent such cardiovascular events [32]. Similarly, specific microbial patterns have been associated with heart failure, suggesting that altering the gut microbiota could help manage heart failure and improve patient outcomes (Figure 2) [33].

Gut Microbiome and Inflammatory Bowel Disease

In IBD, the gut microbiome plays a central role in disease progression and management. Probiotics and prebiotics have been shown to favorably alter the gut microbiota composition, leading to better clinical outcomes in IBD patients. For example, probiotics have significantly improved clinical outcomes, such as a decrease of inflammatory markers like c-reactive protein with an OR −2.45(95%CI: −3.16 to −1.73) and promoting healthy flora in patients with UC increasing the presence of Bifidobacterium and Lactobacillus with an OR: 3.37 (95% CI: 3.28, 3.47) and 2.00 (95% CI: 1.91, 2.09), respectively [34]. On the other hand, prebiotics have not been effective in inducing and maintaining remission in UC reporting OR of 1.05 (95%CI: 0.57 to 1.94), highlighting their need for comparative studies and a better understanding of the gut mechanisms with IBD [35]. Research has shown that probiotics can be as effective as standard treatments for UC, with fewer side effects. Probiotics help maintain remission and reduce relapse rates, supporting their use as a viable option for managing UC [36]. Additionally, microencapsulated sodium butyrate, a microbial fermentation product, has shown promise in reducing inflammatory markers and improving gut health in irritable bowel syndrome, indicating potential benefits for IBD management [37].

Integrative Approach: Microbiome, CVD, and IBD

The interconnection between gut microbiota, CVD, and IBD suggests an integrated approach to managing these conditions could be beneficial. FMT has shown promise in cardiovascular and IBD contexts. FMT can induce significant changes in microbiota composition, leading to metabolic improvements in patients with severe obesity and metabolic syndrome [38]. Similarly, FMT has shown potential therapeutic benefits for IBD patients by inducing changes in microbiota and mucosal gene expression, maintaining remission in UC, and providing a practical approach to managing the disease [39,40]. Future research and clinical trials are essential to explore and validate the therapeutic potential of gut microbiome modulation in both CVD and IBD. By developing targeted interventions to optimize gut microbiota composition, researchers aim to create effective treatments that can be integrated into standard care for cardiovascular health and IBD management. This approach holds promise for transforming healthcare, offering new strategies to prevent and manage these interconnected conditions through the lens of the gut microbiome [41-43].

Diagnostic approaches 

The diagnostic approach to IBD, including Crohn's disease and ulcerative colitis, involves a comprehensive evaluation to confirm the disease, determine its extent, and guide treatment. Key symptoms such as chronic diarrhea, abdominal pain, weight loss, and rectal bleeding prompt further investigation [44,45]. Tests like complete blood count (CBC) for anemia and markers like erythrocyte sedimentation rate (ESR) and C-reactive protein (CRP) assess disease activity and nutritional status [46]. Serologic studies, including perinuclear anti-neutrophil cytoplasmic antibodies (p-ANCA) for ulcerative colitis and anti-Saccharomyces cerevisiae antibodies (ASCA) for Crohn's disease, help differentiate between conditions. Stool studies exclude infections and measure fecal calprotectin, a marker of intestinal inflammation [44,45,47]. Imaging techniques such as endoscopy (colonoscopy, sigmoidoscopy) allow visualization of mucosal inflammation and biopsy for histopathological confirmation [44,45]. Diagnostic imaging such as electrocardiography (ECG), echocardiography, and cardiac MRI provide detailed heart and vasculature assessments. Biomarkers like troponins, B-type natriuretic peptide (BNP), and CRP detect myocardial injury, assess cardiac function, and indicate inflammatory processes. These tools are crucial in detecting and stratifying CVD risk, guiding therapeutic decisions, and monitoring progression [48-50].

Currently, no established guidelines exist for assessing CVD risk, specifically in IBD patients. Typically, IBD patients are young adults who do not qualify for traditional CVD risk assessments. However, the European Society of Cardiology's 2019 guidelines recognized IBD, along with conditions like cancer treatment, rheumatoid arthritis (RA), and systemic lupus erythematosus (SLE), requiring rigorous screening, counseling, and management for atherosclerotic disease. Established guidelines from the European Alliance of Associations for Rheumatology (EULAR) for other chronic inflammatory disorders (CIDs) emphasize assessing disease activity using biomarkers linked to cardiovascular disease and IBD. Elevated inflammatory markers in IBD, such as CRP, IL-6, IL-1, IL-8, and TNF-alpha, predict future cardiovascular events. Diagnostic tools like endoscopic procedures and serologic testing to assess disease activity in IBD can also help in diagnosing/screening for CVD [51-55]. Further research is necessary to develop appropriate strategies for concurrently assessing the risk and diagnosing both IBD and CVD. A summary of the differences between Crohn's and ulcerative colitis is provided in Table 1.

**Table 1:** Comparison of clinical characteristics between Crohn's disease and ulcerative colitis. IBD: inflammatory bowel disease, TNF-α: tumor necrosis factor-alpha, IL1β: interleukin 1 beta, IL-6: interleukin 6, IL-8: interleukin 8, NO: nitric oxide, VWf: Von Willebrand factor, CD40-L: CD40 ligand, SAA: serum amyloid A, LDL: low-density lipoprotein, TMAO: trimethylamine N-oxide, ASCA: anti-Saccharomyces cerevisiae antibodies, p-ANCA: perinuclear anti-neutrophil cytoplasmic antibodies, NOD2/CARD15: nucleotide-binding oligomerization domain 2/caspase recruitment domain family member 15, HLA B-27: human leukocyte antigen B-27, PSC: primary sclerosing cholangitis, CD: Crohn’s disease, CRC: colorectal cancer.

Characteristic	Crohn’s disease	Ulcerative colitis
Location	Any segment of the gastrointestinal tract from mouth to anus, with potential involvement of extra-intestinal sites [1].	Predominantly affects the rectum, extending proximally into the colon. It can involve the entire colon (pancolitis) or be limited to the rectum (proctitis) [56].
Most common site	Terminal ileum and colon (ileocolonic involvement) [1].	Rectum and left-sided colon (sigmoid and descending colon) [56].
Spread	Transmural inflammation with patchy involvement [1].	It features continuous inflammation from the rectum and extends proximally through the colon [56].
Inflammation	Transmural inflammation affects the entire bowel wall thickness [1].	It primarily involves the mucosal and submucosal layers. In severe cases, it may extend into the muscularis propria [56].
Endoscopic findings	Presents with cobblestone appearance, longitudinal ulcers (≥4 to 5 cm), and aphthous ulcerations arranged longitudinally [1].	Edematous mucosa, erythema, loss of vascular markings, mucosal friability, and presence of pseudopolyps in severe disease [56].
Serological marker: ASCA	35% to 50% of Crohn’s disease patients.	1% of ulcerative colitis patients [57].
p-ANCA	Present in 10%-15% of cases [58]	Detected in 60-70% of cases, often correlating with disease severity [59].
Genetic associations	Implicated in multiple genetic loci, including mutations in the NOD2/CARD15 gene, in up to 30% of Crohn’s disease cases. Other genes involved in immune response and epithelial barrier function are also implicated [1].	Associated with HLA B-27 in some cases, indicating genetic predisposition in a subset of patients [56].
Extra-intestinal manifestations	Associated with arthritis, uveitis, and erythema nodosum [1].	Less frequent but may include primary sclerosing cholangitis (PSC), autoimmune disorders, and skin manifestations such as pyoderma gangrenosum and erythema nodosum [56].
Age of onset	Typically, at a younger age (10s to 30s) [1].	Bimodal distribution with peaks in the 20s-30s and 60s-70s [56].
Complications	Fistulas, abscesses, strictures, and anal fissures [1].	Colorectal cancer, severe hemorrhage, and life-threatening toxic megacolon [56].
Prognosis	Mortality risk is increased by up to 50% in CD compared to the general population [60].	On average, the life expectancy of patients is normal.
Gender distribution	Women in Europe and the USA are affected approximately twice as often as men by Crohn’s disease, in Asia more men are affected than women [61].	Male sex is associated with a higher incidence rate of ulcerative colitis compared with female sex (12.8 versus 8.8 cases per 100,000 person-years [61].
Risk of colorectal cancer	The risk of CRC and small bowel cancer is increased two- to eightfold among IBD patients [60].	
Cardiovascular association	19% increase in their risk of developing heart failure up to 20 years after diagnosis, regardless of what type of IBD the patient had[8].	

Recent research has provided significant insights into the liver enzyme profiles of patients with UC and Crohn's disease. Studies have aimed to characterize the elevations of specific liver enzymes to avoid confusion with other diseases that also elevate liver enzymes and their relationship with long-term outcomes [62-65]. It has been observed that both UC and Crohn's disease can lead to elevated levels of alkaline phosphatase (ALP). However, the elevation of liver enzymes is more prevalent among Crohn's disease patients. Notably, this elevation has been associated with an increased impact on mortality outcomes in these patients. While more research is necessary to generalize these findings, the current evidence underscores the importance for clinicians to consider these biochemical values. This comprehensive assessment can aid in the holistic management of patients with inflammatory bowel diseases, ensuring that liver enzyme abnormalities are appropriately addressed [64-66].

Therapeutic strategies

The treatment landscape for IBD includes several medication classes aimed at managing symptoms and achieving remission [67]. Foundational therapies like aminosalicylates (5-aminosalicylic acid, sulfasalazine) are effective in UC for reducing relapse rates and potentially lowering colorectal cancer risk, but their efficacy in CD is debated. Corticosteroids like prednisone are used for inducing remission during flares but lack efficacy in maintaining remission. Immunomodulators, including thiopurines (azathioprine, 6-mercaptopurine, and 6-thioguanine), methotrexate, calcineurin inhibitors (cyclosporine A, tacrolimus), and Janus kinase (JAK) inhibitors, target immune function to control inflammation. Thiopurines reduce hospitalization and surgery rates in IBD, while calcineurin inhibitors have potent immunosuppressive effects but notable adverse effects like renal damage. Biologics represent a significant advancement, especially for refractory cases. Anti-TNF-α monoclonal antibodies (infliximab and adalimumab) reduce inflammation in moderate to severe UC and lower colectomy rates, whereas newer biologics (ustekinumab and vedolizumab) show efficacy in both UC and CD, although they can cause primary or secondary loss of response and require monitoring. Emerging therapies like S1P receptor modulators (ozanimod) are primarily for non-responsive cases, although their long-term efficacy and safety profiles are still being evaluated. Overall, personalized treatment strategies are needed based on disease type, severity, and individual response to balance efficacy while minimizing side effects [67]. The microbiome's role in IBD is pivotal, influencing inflammation, therapy response, and disease recurrence risk. Modulating gut microbiota by enhancing anti-inflammatory bacteria through probiotics and prebiotics. Synbiotics help alleviate symptoms and promote healing. Conversely, inflammatory bacteria can be targeted with antibiotics or phage therapy to reduce their abundance. Fecal microbiota transplantation offers a microbiome reset, showing promise in refractory cases. Dietary interventions like low-carb and high-fiber diets affect microbiota composition and may lower IBD risk, emphasizing the microbiome as a crucial therapeutic target needing further study. Physical activity's impact varies; while it may protect against CD, its effect on UC risk is inconclusive [68]. 

The interplay between gut microbiota and CVD reveals that dietary choices significantly shape microbiota composition, affecting disease development. For instance, certain foods metabolized by gut bacteria into TMAO correlate with increased CVD risk. PAMPs from gut bacteria contribute to vascular inflammation and atherosclerosis. Physical activity is recognized for its beneficial effects on CVD and gut microbiota. Overall, adopting a healthy diet rich in unsaturated fatty acids, fruits, and vegetables is advised for preventing and managing diseases influenced by gut microbiota [69]. Antihypertensive medications also interact with gut microbiota, potentially influencing treatment outcomes. Drugs like diuretics, β-blockers, calcium channel blockers, and ACE inhibitors induce specific changes in gut bacteria, affecting blood pressure regulation. Evidence from studies in hypertensive rats suggests that drugs like captopril and losartan exert their effects partially through interactions with gut microbiota. These interactions highlight a complex relationship and the need for personalized treatment strategies based on individual microbiome profiles to optimize therapeutic outcomes [70]. 

Interdisciplinary management and preventive strategies 

Interdisciplinary work is the fundamental axis for adequate management of the IBD-CV relationship. In addition to the timely diagnosis and management of these pathologies, secondary prevention should be a priority. In this regard, it is important to note that there are guidelines made available by the American Heart Association to reduce the risk of cardiovascular events in patients with IBD, which, in addition to a multidisciplinary approach, include the management of disease remission, which is an important potentiator of the risk of Atherosclerotic Cardiovascular Disease (ASCVD). It also highlights the importance of aggressive reduction of cardiovascular risk factors through screening with blood pressure, glucose, lipid panel, and lifestyle changes, as previously mentioned. Finally, the health professional is urged to implement the ACC/AHA guidelines for risk assessment and management in patients with chronic inflammatory conditions [71]. On the other hand, it is important to emphasize that personalized preventive strategies should be developed for people with IBD since by identifying those at risk of developing complications, healthcare providers can guide interventions, encouraging shared and informed decision-making. A hot example of this is the potential of coronary calcium scoring for the preventive cardiologist, whereby useful cross-sectional studies such as MRI or coronary CT scans can be used to assess anticipated risks of atherosclerotic events [72].

In general, the management of chronic degenerative diseases requires patient education. It is considered fundamental in the prevention of cardiovascular disease complications. Still, in the case of IBD, a recent systematic review of the Cochrane database concludes that there is possibly no additional benefit over medication and usual care in these patients, considering the difficulty in determining whether the level of education or access to health care influences each individual. Still, this requires careful interpretation and further research [73]. Despite this, the levels of anxiety and depression in these patients could be improved by being informed of the course of the disease and the appropriate management they require to avoid complications may be reduced [74].

Research gaps and future directions 

Current limitations regarding the IBD-CVD link lie in possible treatments, underlying molecular mechanisms, and diagnosis by laboratory aids such as biomarkers that can predict pathological mechanisms and cardiovascular risk [75]. Concerning possible treatments, there are encouraging studies that show improvement and even remission of ulcerative colitis through microbial fecal transplantation, which should be studied to determine if there is a benefit at the cardiovascular level [76,77]. It will also be important to delve into the study of small-molecule antimicrobial enzyme therapies, which have had positive results in inhibiting the formation of atherosclerotic plaque in mice, but still have limitations [67,78]. Furthermore, it should be taken into account that some immunosuppressive treatments used in IBD have different effects on cardiometabolic function, both beneficial in the case of aminosalicylates or anti-TNF-alpha and adverse in the case of corticosteroids so that interdisciplinary management should be optimized to provide personalized treatment depending on the cardiovascular function of each patient [14]. However, large-scale studies are indispensable to consolidate the evidence presented so far [79]. In the last decade, there has been evidence that reduced levels of trimethylamine N-oxide are observed in patients with IBD, so it can be considered an important biomarker [80]. However, its usefulness goes beyond this, as some studies suggest that a therapy aimed at its inhibition would represent a therapeutic potential for cardiometabolic diseases [78]. The reality is that there are still obstacles to consolidating its therapeutic use since the human microbiome is not sufficiently understood to direct therapies at this level, and more studies are needed to increase pathophysiological understanding. Aware of the situation in the medical community, it is crucial to address these challenges to advance research on therapeutic applications [81].

## Conclusions

This review underscores the complex interplay between IBD and CVD, emphasizing the pivotal roles of inflammation and the gut microbiome. While the current evidence-primarily highlights indirect associations, these insights provide a foundation for future research and potential clinical applications. Modulating gut microbiota through dietary interventions, probiotics, and FMT represents promising research avenues, though their direct clinical relevance requires further validation through rigorous studies. Moreover, the importance of interdisciplinary management is highlighted, advocating for regular cardiovascular risk assessments and personalized preventive strategies tailored to this unique patient population. Future research should prioritize elucidating the precise molecular mechanisms linking IBD and CVD, identifying reliable biomarkers for early detection, and developing innovative therapeutic approaches that concurrently address both gut and cardiovascular health. These efforts will be crucial in translating our current understanding into concrete clinical benefits, ultimately improving outcomes for patients with IBD and associated cardiovascular risks.
